# Effects of customized corneal cross‐linking on higher‐order aberrations in progressive keratoconus and low‐grade myopia

**DOI:** 10.1111/aos.17432

**Published:** 2024-12-19

**Authors:** Jad Hayek, Andreas Viberg, Sofie Elving, Anneli Fredriksson, Anders Behndig

**Affiliations:** ^1^ Department of Clinical Sciences/Ophthalmology Umeå University Umeå Sweden

**Keywords:** cross‐linking, higher‐order aberrations, keratoconus, myopia, visual acuity

## Abstract

**Objective:**

To evaluate the effects of customized corneal collagen cross‐linking (CXL) on higher‐order aberrations (HOAs) in keratoconus (KC): vertical coma (VC), horizontal coma (HC), spherical aberration (SA), trefoil (TF) and astigmatism, compared with the same effects in healthy eyes undergoing CXL for low‐grade myopia.

**Methods:**

This mixed‐designed study included 38 eyes of 38 patients with KC, treated and followed prospectively, who received customized epi‐on CXL in high oxygen, and a retrospective control group of 23 eyes from 23 patients who underwent central 4‐mm CXL treatment for low‐grade myopia. VC, HC, SA, TF and keratometry values were obtained from Pentacam HR® measurements at baseline and at 1, 6, 12 and 24 months post‐treatment. Statistical analyses included paired T‐tests for changes over time and Pearson correlation tests to assess relationships between aberrations, best spectacle‐corrected and low‐contrast visual acuities (BSCVA and LCVA, respectively) and CXL parameters.

**Results:**

Reduced HOAs and improved visual acuities were observed in KC. A 20% reduction in VC was observed at 24 months (from −1.82 ± 1.15 μm to −1.46 ± 1.01 μm; 95% CI: [0.155, 0.629], *p* = 0.002), while a 17% reduction in HC was observed at 12 months (from −0.35 ± 0.56 μm to −0.29 ± 0.62 μm; 95% CI: [0.003, 0.096], *p* = 0.037). A positive correlation was found between baseline VC and the level of improvement in VC at 24 months (*R* ^2^ = 0.200, *p* = 0.015). SA increased by 126% at 24 months (from −0.21 ± 0.62 μm to 0.054 ± 0.52 μm; 95% CI: [0.143, 0.347], *p* ≤ 0.001). TF and astigmatism did not alter from the treatment. In myopia, the natural positive SA increased by 57% post‐treatment (from 0.14 ± 0.061 μm to 0.22 ± 0.076 μm at 24 months; 95% CI: [0.067, 0.098], *p* ≤ 0.001), while changes in VC and HC were minor and BSCVA remained stable.

**Conclusion:**

Customized CXL effectively reduces HOAs in KC. For VC the improvement is larger in cases with higher preoperative VC, indicating that the concept of customization has its intended effect. Accordingly, SA and visual acuities improve in KC whereas CXL for low‐grade myopia tends to increase corneal SA unfavourably.

## 1 INTRODUCTION

Keratoconus (KC) is a progressive ectatic corneal disease characterized by central or paracentral thinning of the cornea, which gives rise to irregular refractive errors and visual deterioration (Rabinowitz, 1998). The underlying mechanisms of KC are multifaceted, involving molecular genetics, enzymology, proteomics and biomechanics that all influence disease progression and severity (Davidson et al., 2014). Although the exact aetiology of KC remains unclear, it is known that localized thinning and consequent biomechanical weakening are key drivers of ectasia and the formation of a cone‐shaped cornea (Blackburn et al., 2019).

The progressive corneal distortion in KC leads to the development of higher‐order aberrations (HOAs), which cannot be corrected with sphero‐cylindrical spectacles, and which vastly supersede the minute aberrations of a normal cornea. These HOAs contribute to deterioration of retinal image quality and to the reduced visual performance in keratoconus (Applegate et al., 2000; Erdinest et al., 2024). Corneal aberrometric analysis has been suggested as a method for grading keratoconus and for early diagnosis on clinical suspicion (Alio & Shabayek, 2006).

The main higher‐order aberration in keratoconus is coma, in particular vertical coma (Erdinest et al., 2024). Changes in horizontal coma, trefoil and spherical aberration can also be seen to a variable degree in KC, depending on the size and localization of the keratoconus cone (Alio & Shabayek, 2006; Erdinest et al., 2024). A healthy cornea has a positive spherical aberration, which in younger individuals is compensated for by a negative spherical aberration in the crystalline lens (Applegate et al., 2000). Treatments that flatten the central cornea in a healthy eye (e.g. older protocols for myopic excimer laser ablation) increase the positive spherical aberration of the cornea, which can have a negative impact on the quality of vision (Applegate et al., 2000). In KC the corneal spherical aberration is generally lower than in a healthy cornea (i.e. less positive or more negative), especially if the cone is located centrally (Applegate et al., 2000; Erdinest et al., 2024). Central flattening of the cornea in such cases can shift the corneal spherical aberration in the positive direction, which can have a beneficial impact on the quality of vision (Applegate et al., 2000; Erdinest et al., 2024).

Corneal collagen cross‐linking (CXL) is the mainstay of treatment in progressive keratoconus, and its chief aim has been to halt disease progression by increasing the biomechanical strength of the corneal tissue through strengthening the inter‐ and intrafibrillar stromal bonds (Spoerl et al., 2011). The standard CXL technique involves removal of the epithelium (epi‐off CXL), but CXL can also be performed with the epithelium intact (transepithelial or epi‐on CXL). Epi‐on CXL aims to achieve disease stability while reducing post‐procedural pain, risk of infection, haze and other complications (Rush & Rush, 2017). An intact epithelium may, however, reduce the effectiveness of the procedure by acting as a barrier to riboflavin and oxygen diffusion into the corneal stroma (Elving et al., 2024). The latter can be compensated for by adding oxygen to the ambient air over the cornea during the CXL treatment (Elving et al., 2024).

A CXL treatment can also be customized and adapted to the individual corneal shape, by delivering higher energies to the most protruding part or the cornea (Elving et al., 2024). Theoretically, such a regimen has the potential to regularize the corneal shape and reduce the corneal HOAs to a higher degree than a standard CXL treatment. Some studies have compared the change in corneal HOAs between different CXL treatment protocols (Kirgiz et al., 2019; Naslund et al., 2022; Singal et al., 2020). In the present study, we evaluated the effects on corneal HOAs after CXL treatment for KC using a customized epi‐on high‐oxygen protocol. Our analyses focused on changes in vertical coma (VC), horizontal coma (HC) and spherical aberration (SA) obtained with rotating Scheimpflug camera measurements (Pentacam HR®, Oculus, Inc.). As a control the corresponding HOAs were analysed after epi‐on high‐oxygen central 4‐mm CXL treatment for low‐grade myopia in healthy eyes.

## 2 MATERIALS AND METHODS

This was a mixed‐design study including 38 eyes from 38 patients with KC, who received customized epi‐on CXL in high oxygen, and a retrospectively collected control group of 23 eyes from 23 patients who had undergone central 4‐mm CXL treatment for low‐grade myopia. One eye from each patient was chosen at random in both groups, and the data from left eyes were mirror‐imaged to make them comparable with right eyes.

The group sizes were determined by two power analyses. To determine the sample size of the KC group we assumed that vertical coma (VC) has a major impact on the reduction in visual quality in keratoconus (Naderan et al., 2018), and that a reduction in this aberration would be an important factor in improving the visual quality after customized CXL. Based on a standard deviation of the pre‐ to post‐treatment difference in VC of 0.7 (taken from our previous studies with the same equipment) a sample size of 22 (38, with 20% of cases lost to follow‐up) could detect a reduction in VC of 0.43 (15%) with 80% probability at alpha = 0.05. Thus, 38 eyes from 38 subjects with KC were included.

For the control group of low‐grade myopes, data were retrospectively collected from our previously published studies (Fredriksson et al., 2020; Naslund et al., 2021), and the group size was determined by a power analysis of the change in spherical aberration (SA). With a standard deviation of the pre‐ to post‐treatment difference in SA of 0.04 a sample size of 18 (23, with 20% lost to follow‐up) could detect an increase in spherical aberration of 0.028 (20%) with 80% probability at alpha = 0.05. Thus, 23 eyes from 23 subjects with a complete set of 3 Pentacam HR® measurements of good quality at a maximum number of visits were randomly selected. The control group matched the KC group for age (24.8 ± 5.1 vs. 26.3 ± 4.5 years, *p* = 0.28), for uncorrected visual acuity (UCVA: 0.59 ± 0.57 LogMAR vs. 0.59 ± 0.21 LogMAR, *p* = 0.98) and for the degree of myopia (Spherical Equivalent: −1.76 ± 2.22 D vs. −1.41 ± 0.44 D, *p* = 0.35), but not for gender (males/females 33/5 for KC and 10/13 for controls, *p* < 0.001).

The total corneal HOA:s from a 6‐mm central corneal area were extracted from Zernike polynomials generated by the built‐in ray tracing algorithm of Pentacam HR®. Changes in VC, horizontal coma (HC), SA, trefoil 30° (TF30°) and trefoil 0° (TF0°) from pre‐treatment baseline values were registered at four timepoints following treatment: 1 month, 6 months, 12 months, and 24 months. For all HOA values presented, the means of three Pentacam HR® measurements were used. For astigmatism, the simulated K‐values K1 and K2 were retrieved from the Pentacam HR® measurements and the net cylinder magnitude (M; K2 − K1) and axis, α, were used in formulas from Naeser (Naeser, 2008) to calculate two astigmatic power vectors; the meridional power/polar value in 0 degrees, KP(0), and the torsional power/polar value in 45 degrees KP(45):

$$\mathrm{KP}\left(0\right)=M\left(\cos2\alpha \right)$$

$$\mathrm{KP}\left(45\right)=M\left(\sin2\alpha \right)$$

The mean change in astigmatism from the treatments was calculated from KP(0) and KP(45) and then re‐converted back to cylindrical values with the formula $M=\sqrt{{\left(\mathrm{KP}0\right)}^{2}+{\left(\mathrm{KP}45\right)}^{2}}$. In addition, the astigmatic change was calculated by simple subtraction. Paired *t*‐tests were performed to assess the changes in HOAs and astigmatism over time. A Pearson correlation test was conducted to assess the relationships between HOAs, measures of high‐ and low‐contrast visual acuities and treatment parameters, specified below.

Visual acuities were assessed with a standard logMAR chart using the ETDRS‐fast protocol (Camparini et al., 2001) at the above timepoints, including best spectacle‐corrected visual acuity (BSCVA) for both keratoconus (KC) and myopia. Additionally, low‐contrast visual acuity (LCVA) was evaluated for KC at 10% and 2.5% contrast. A p‐value of <0.05 was considered statistically significant for all analyses.

Nine patients (24%) of the KC group did not attend their 24‐month visit, and one patient (3%) was missing at each of the 1–12‐month visits. LCVA 2.5% was too low to measure in two cases (5%) at baseline and in one case (3%) at 1 month. In the myopia group, five patients (28%) did not attend their 24‐month visit.

Effect sizes for between‐group differences were assessed with Cohen's *d* at all timepoints for VC, HC, SA and BSCVA, and a post‐hoc power analysis at α=0.05 was performed for variables where between‐group comparisons were done.

All treatments were conducted at Umeå University Hospital. The study adhered to the tenets of the Declaration of Helsinki and was approved by the Swedish Ethical Review Authority. Prior to inclusion, written informed consent was obtained from all participants.

### 2.1 Treatment protocols

The cross‐linking protocols have been detailed previously (Elving et al., 2024; Naslund et al., 2022).

The treatment was started by applying three drops of tetracaine 1%, followed by a lid speculum and an oxygen applicator mask (Glaukos, Corp.). The corneal epithelium was not removed, but gently wiped with a cellulose sponge soaked with a HPMC‐based riboflavin solution (ParaCel Part 1; 0.25%, riboflavin ophthalmic solution, Glaukos, Corp.) to remove the mucin coating of the corneal surface. ParaCel Part 1 was then repeatedly added to completely cover the cornea at 90 s intervals for 4 min, whereafter ParaCel Part 2 (0.22%, riboflavin ophthalmic solution, Glaukos, Corp.) was given at the same rate for a total of 6 min. A few drops of saline 0.9% was used to rinse the corneal surface from excess riboflavin before illumination to avoid UV‐A shielding. The oxygen applicator mask provided humidified 100% oxygen at a flow rate of 2.5 L/min starting 2 min prior to UV‐illumination and was continued until completed UV‐illumination. To confirm a sufficiently oxygenated atmosphere around the eye, a Model 901 oxygen meter device (Quantek Instruments, Grafton) was used to confirm ≥90% oxygen before and immediately after illumination.

The individual treatment design for keratoconus eyes was customized according to the individual topography assessed by Pentacam HR®, where the maximum dose given was determined by *K*_Max_:*K*_max_ <45 D was given 7.2 J/cm^2^; *K*_Max_ 45–50 D was given 10 J/cm^2^, and *K*_Max_ >50 D was given 15 J/cm^2^. The treatment patterns were designed using the Mosaic device system (Avedro/Glaukos Mosaic system, Glaukos Corp.) utilizing a posterior corneal surface map exported from the Pentacam HR®, with the maximum effect at the steepest point and elliptical zones with a tapering of 2 Joules (J) per 2 D of reduced steepness towards the corneal periphery. Elliptical zones were chosen since they were found to match the form of the zones of equal power on the posterior corneal surface in keratoconus most closely. A 6 mm vertex‐centred circular zone was treated with 5.4 J/cm^2^ in all keratoconus eyes to render a more generalized moderate‐level cross‐linking effect in the central cornea. Pulsed UV‐light was used in all treatments. The treatment power of the 365 nm pulsed UV‐A light was 30 mW/cm^2^ and to achieve a constant total treatment time of 16 minutes and 40 seconds, the pulse settings for the different treatment effects were: 1 second on / 1 second off for 15 J/cm^2^; 0.5 seconds on / 1 second off for 10 J/cm^2^, and 0.4 seconds on / 1.2 seconds off for 7.2 J/cm^2^.

In the treatments of low‐grade myopia used in the control group, a vertex‐centred 4.0 mm annular zone with a central 2.0 mm sparing was treated at a total fluence of 15 J/cm^2^, pulsed UV‐light 1 second on / 1 second off, with a total treatment time of 16 minutes and 40 seconds in all cases.

Minute eye movements during the treatment were compensated for by the eye tracker of the Mosaic System. No additional riboflavin was applied during the treatments. One or two drops of saline 0.9% were given if the cornea became too dry for the eye tracker to detect the pupil border, and one or two drops of tetracaine 1% were added if the participant reported discomfort.

The treatment fields were characterized in the calculations by their spatial coordinates and the applied energy in J/cm^2^. The parameters *X*_Max_ and *Y*_Max_ were calculated as the product of the maximum delivered energy and the respective X‐ and Y‐coordinates of the centre of the zone of maximum delivered energy, and ‘Distance to Max’ was calculated as the Euclidean norm of the latter parameters, given by $\sqrt{{\left(Y_{\mathrm{Max}}\right)}^{2}+{\left(X_{\mathrm{Max}}\right)}^{2}}$. Thus, Distance to Max is the distance from the corneal vertex to the centre of the zone of maximum delivered energy.

## 3 RESULTS

**FIGURE 1:**
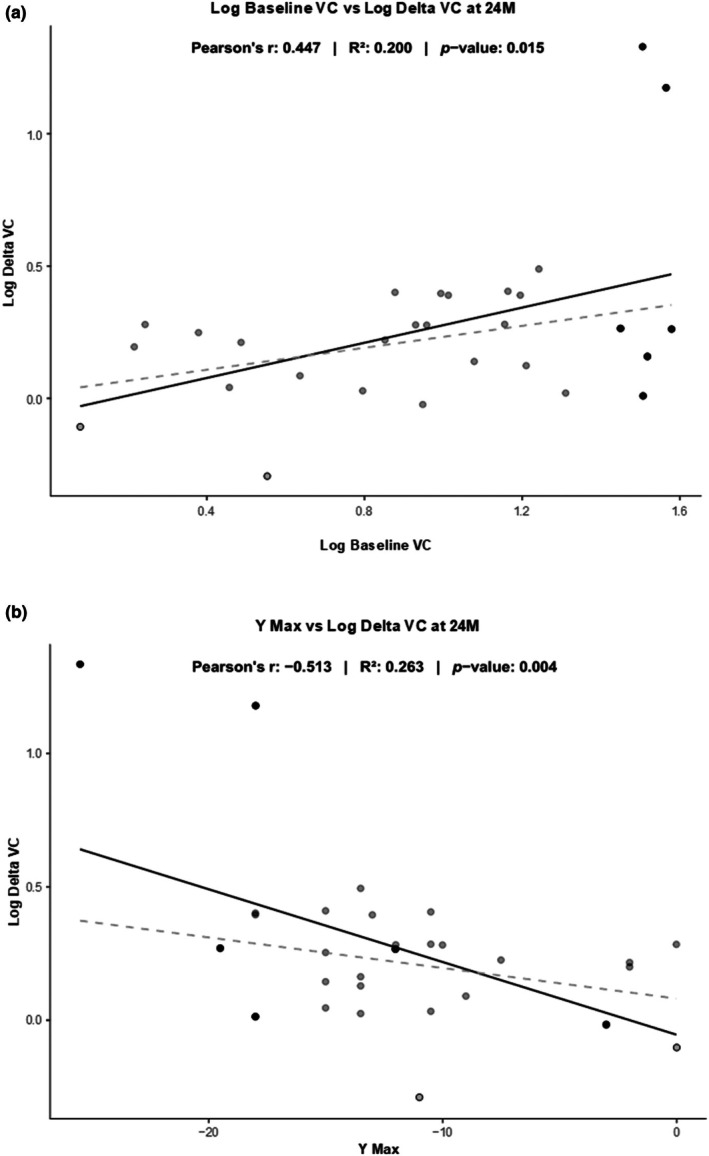
The plots show ∆ vertical coma (VC) in relation to baseline VC (a, above) (Pearson's *r*: 0.447 – *R* ^2^: 0.200 – *p* = 0.015) and *Y*_Max_ (b, below) (Pearson's *r*: −0.513 – *R* ^2^: 0.263 – *p* = 0.004). (a) Logarithmic correlation between baseline VC and ∆ VC at 24 M. (b) Logarithmic correlation between *Y*_Max_ and ∆ VC at 24 M. The dashed line represents the relationship between the variables in the absence of the six largest baseline VCs, also with among the largest improvements in VC.

In KC, significant decreases in HOAs were observed over time following treatment. The largest improvement was seen in the pronounced negative VC 20% improvement at 24 months (from −1.82 ± 1.15 μm to −1.46 ± 1.01 μm; 95% CI: [0.155, 0.629]). A gradual increase was seen throughout the follow‐up (Table 1). Interestingly, a significant correlation was found between the level of pre‐treatment VC and the level of improvement at all timepoints (*R* ^2^ = 0.200; *p* = 0.015 at 24 months). In addition, the improvement in VC was also significantly inversely correlated to *Y*_Max_ (*R* ^2^ = 0.270; *p* = 0.004 at 24 months). Despite the improvement in VC, it was still significantly lower than the VC seen in healthy corneas at 24 months (post‐hoc power analysis at 24 months 100% at α = 0.05; Cohen's *d* = 1.4; *p* < 0.001; Table 1). As we noted a possible influence on the relationship between ∆ VC, baseline VC, and *Y*_Max_ by a few cases with large improvements in VC, we decided to carry out a Log‐transformation of these values. The correlations remained significant at 24 months also following Log‐transformation (Figure 1). The correlations were significant also without the six highest baseline VC at 12 months using both Pearson and Spearman correlation tests, but not at 24 months, possible due to the dropout of study participants at 24 months.

**TABLE 1:** Pre‐ and post‐treatment values for keratoconus (KC) and myopia.

Higher‐order aberration/measure of visual acuity $\bar{x}$ (*σ*)	Baseline		1 M		6 M		12 M		24 M	
	KC	Myopia	KC	Myopia	KC	Myopia	KC	Myopia	KC	Myopia
Vertical coma (μm)	−1.80	−0.027	−1.61	−0.084	−1.60	−0.025	−1.54	−0.042	−1.46	−0.050
	(1.15)	(0.169)	(1.06)	(0.164)	(1.05)	(0.156)	(1.04)	(0.168)	(1.01)	(0.182)
95% CI			[0.106, 0.280]	[−0.090, −0.023]	[0.097, 0.297]	[−0.031, 0.035]	[0.089, 0.374]	[−0.051, 0.021]	[0.155, 0.629]	[−0.069, 0.000]
*p* value			**<0.001**	**0.002**	**<0.001**	0.910	**0.002**	0.400	**0.002**	0.050
Cohen's *d*	1.4		1.4		1.5		1.4		1.4	
Horizontal coma (μm)	−0.345	−0.068	−0.370	−0.060	−0.329	−0.041	−0.291	−0.034	−0.294	−0.031
	(0.558)	(0.121)	(0.594)	(0.125)	(0.540)	(0.097)	(0.587)	(0.085)	(0.620)	(0.075)
95% CI			[−0.117, 0.012]	[−0.016, 0.033]	[−0.041, 0.072]	[−0.003, 0.056]	[0.003, 0.096]	[0.001, 0.067]	[−0.008, 0.122]	[0.008, 0.079]
*p* value			0.109	0.471	0.581	0.072	**0.037**	**0.044**	0.085	**0.019**
Cohen's *d*	0.5		0.5		0.5		0.3		0.4	
Spherical aberration (μm)	−0.208	0.137	−0.041	0.133	−0.048	0.220	0.021	0.226	0.054	0.219
	(0.621)	(0.061)	(0.482)	(0.116)	(0.611)	(0.080)	(0.509)	(0.076)	(0.520)	(0.077)
95% CI			[0.099, 0.237]	[−0.050, 0.041]	[0.089, 0.231]	[0.062, 0.103]	[0.114, 0.254]	[0.070, 0.107]	[0.143, 0.347]	[0.067, 0.098]
*p* value			**<0.001**	0.852	**<0.001**	**<0.001**	**<0.001**	**<0.001**	**<0.001**	**<0.001**
Cohen's *d*	0.7		0.6		0.6		0.5		0.5	
BSCVA LogMAR	0.109	−0.057	0.083	−0.037	0.010	−0.081	0.002	−0.077	0.006	−0.066
	(0.217)	(0.063)	(0.206)	(0.048)	(0.136)	(0.057)	(0.161)	(0.056)	(0.107)	(0.059)
95% CI			[−0.045, 0.019]	[−0.005, 0.045]	[−0.125, −0.049]	[−0.055, 0.008]	[−0.150, −0.054]	[−0.051, 0.013]	[−0.177, −0.057]	[−0.055, 0.031]
*p* value			0.421	0.108	**<0.001**	0.134	**<0.001**	0.228	**<0.001**	0.558
Cohen's *d*	0.7		0.6		0.6		0.5		0.5	
Missing (*n* =)	0	0	1	0	1	0	1	0	9	5
LCVA 10% LogMAR	0.481	–	0.373	–	0.330	–	0.278	–	0.297	–
	(0.436)		(0.223)		(0.220)		(0.218)		(0.150)	
95% CI			[−0.173, 0.007]	–	[−0.226, −0.028]	–	[−0.262, −0.081]	–	[−0.303, −0.051]	–
*p* value			0.071	–	**0.014**	–	**<0.001**	–	**0.008**	–
Missing (*n* =)	0	–	1	–	1	–	1	–	9	–
LCVA 2.5% LogMAR	1.187	–	0.989	–	0.890	–	0.792	–	0.816	–
	(1.032)		(0.780)		(0.740)		(0.657)		(0.716)	
95% CI			[−0.423, 0.063]	–	[−0.645, −0.030]	–	[−0.707, −0.084]	–	[−0.784, −0.025]	–
*p* value			0.142	–	**0.032**	–	**0.014**	–	**0.037**	–
Missing (*n* =)	2	–	2	–	1	–	1	–	9	–

*Note*: Values in bold are statistically significant.

Abbreviations: BSCVA, best spectacle‐corrected visual acuity; KC, keratoconus; LCVA, low‐contrast visual acuity.

A less pronounced improvement in HC −17% – was found at the 12‐month follow‐up – from −0.35 ± 0.56 μm to −0.29 ± 0.62 μm (95% CI: [0.003, 0.096], *p* = 0.037). The significance of the reduction did not persist to 24 months, which may be due to the dropout of study participants (Table 1). No significant correlation was discernible between HC and *X*_Max_ (Table 2).

**TABLE 2:** Pearson correlation coefficients between visual and treatment parameters.

Correlation	*r* (*R* ^2^)			
	1 M	6 M	12 M	24 M
∆ VC//*Y*_Max_	−0.275 (0.076)	−0.345 (0.119)	−0.424 (0.180)	−0.520 (0.270)
*p*‐value	0.094	**0.034**	**0.009**	**0.004**
∆ HC//*X*_Max_	−0.033 (0.001)	−0.132 (0.017)	0.114 (0.013)	0.061 (0.004)
*p*‐value	0.843	0.428	0.500	0.753
∆ SA//Distance to Max	−0.184 (0.034)	0.005 (0.000)	−0.018 (0.000)	−0.118 (0.014)
*p*‐value	0.270	0.974	0.914	0.541
∆ BSVCA//*X*_Max_	−0.144 (0.021)	−0.042 (0.002)	0.002 (0.000)	0.010 (0.000)
*p*‐value	0.396	0.806	0.992	0.962
∆ LCVA 10%//*X*_Max_	−0.023 (0.001)	0.060 (0.004)	−0.085 (0.007)	0.031 (0.001)
*p*‐value	0.892	0.729	0.616	0.876
∆ LCVA 2.5%//*X*_Max_	0.228 (0.052)	0.250 (0.062)	0.185 (0.034)	0.219 (0.048)
*p*‐value	0.182	0.148	0.280	0.273
∆ BSVCA//*Y*_Max_	0.166 (0.028)	0.140 (0.020)	0.042 (0.002)	0.090 (0.008)
*p*‐value	0.327	0.446	0.806	0.656
∆ LCVA 10%//*Y*_Max_	−0.120 (0.014)	−0.131 (0.017)	−0.023 (0.001)	−0.025 (0.001)
*p*‐value	0.480	0.446	0.895	0.902
∆ LCVA 2.5%//*Y*_Max_	0.253 (0.064)	0.332 (0.110)	0.349 (0.122)	0.432 (0.187)
*p*‐value	0.137	0.051	**0.034**	**0.025**
∆ BSVCA//Distance to Max	−0.163 (0.027)	−0.118 (0.014)	−0.023 (0.001)	−0.072 (0.005)
*p*‐value	0.335	0.487	0.893	0.721
∆ LCVA 10%//Distance to Max	0.139 (0.019)	0.144 (0.021)	0.059 (0.003)	0.052 (0.003)
*p*‐value	0.412	0.394	0.731	0.796
∆ LCVA 2.5%//Distance to Max	−0.249 (0.062)	−0.328 (0.108)	−0.334 (0.112)	−0.417 (0.174)
*p*‐value	0.142	0.055	**0.043**	**0.030**

*Note*: Values in bold are statistically significant.

Abbreviations: BSCVA, best spectacle‐corrected visual acuity; KC, keratoconus; LCVA, low‐contrast visual acuity.

SA was negative in KC before treatment but became gradually more positive post‐treatment, that is, approaching but not reaching the values seen in healthy corneas (post‐hoc power analysis pre‐treatment 93% at α = 0.05; Table 1). At 24 months, a 126% increase was observed in SA – from −0.21 ± 0.62 μm to 0.054 ± 0.52 μm (95% CI: [0.143, 0.347], *p* ≤ 0.001). No significant association was found between reduction in SA and Distance to Max (Table 2).

In myopia, VC and HC were generally small, and the changes seen after treatment were significant in some cases but likely of little clinical relevance (Table 1). In contrast, the positive SA seen before treatment in healthy myopes increased further by 57% after treatment – from 0.14 ± 0.061 μm to 0.22 ± 0.076 μm at 24 months (95% CI: [0.067, 0.098], *p* ≤ 0.001; Table 1).

The trefoil values at baseline in KC deviated less from the control group than VC and SA (0.16 ± 0.36 μm vs. −0.038 ± 0.12 μm; Cohen's *d* 0.5; *p* = 0.003, and 0.079 ± 0.25 μm vs. 0.029 ± 0.058 μm; Cohen's *d* 0.2; *p* = 0.24 for trefoil 30° and 0°, respectively). Furthermore, the trefoil values did not change significantly from the treatment for KC or controls – at 24 months the corresponding values were 0.10 ± 0.29 μm (*p* = 0.067) and −0.065 ± 0.057 μm (*p* = 0.78) and 0.024 ± 0.39 μm and 0.011 ± 0.068 μm (*p* = 0.38) for trefoil 30° and 0°, respectively.

As expected, the K‐values were higher in KC than in controls at baseline −45.57 ± 2.57 versus 43.63 ± 1.45 D; post‐hoc power analysis 96% at α = 0.05; Cohen's *d* 0.7; *p* < 0.001. The astigmatism in KC did not change significantly from the treatment: 2.71 ± 1.82 D at baseline versus 2.60 ± 1.81 D at 24 months, *p* = 0.30. Calculating the change in astigmatism using power vectors according to Naeser (Naeser, 2008), however, the astigmatic change in KC was 0.53 ± 0.44 D in KC versus 0.21 ± 0.14 D in controls (post‐hoc power analysis at 24 months 96% at α = 0.05; Cohen's *d* 0.7; *p* < 0.001).

Significant improvements in BSCVA, LCVA 10% and LCVA 2.5% were seen throughout the follow‐up for KC (Table 1). BSVCA increased by 94%, from 0.109 ± 0.217 LogMAR to 0.006 ± 0.107 LogMAR (95% CI: [0.057, 0.177], *p* ≤ 0.001); LCVA 10% increased by 38%, from 0.481 ± 0.536 LogMAR to 0.297 ± 0.150 LogMAR (95% CI: [0.051, 0.303], *p* = 0.008) and LCVA 2.5% increased by 30%, from 1.187 ± 1.032 to 0.816 ± 0.716 (95% CI: [0.025, 0.784], *p* = 0.037), respectively, at 24 months. In the myopia group, BSCVA remained stable throughout the follow‐up (Table 1).

In KC, correlation analyses of the improvements in visual acuities and the different treatment parameters revealed an association between the improvement in LCVA 2.5% at 24 months and *Y*_Max_ (*R* ^2^ = 0.187; *p* = 0.025; Table 2). Moreover, the improvement in LCVA 2.5% at 24 months was inversely related to Distance to Max (*R* ^2^ = 0.174; *p*‐value = 0.030; Table 2).

Effect sizes for between‐group differences assessed with Cohen's *d* are shown in Table 1 at all timepoints for variables where between‐group differences were assessed.

## 4 DISCUSSION

Higher‐order aberrations (HOAs) significantly impair visual quality and performance in keratoconus (KC). It has been demonstrated that KC eyes exhibit up to 5.5 times higher levels of HOAs compared to normal eyes, with vertical coma accounting for more than half of the total HOAs (Pantanelli et al., 2007). The corneal cross‐linking (CXL) procedure is known not only to halt disease progression but also to enhance visual acuity and quality by reducing HOAs (Greenstein et al., 2012; Naderan & Jahanrad, 2017; Wisse et al., 2016).

The customized CXL approach in the present study demonstrates a more pronounced effect on HOAs, particularly VC, when preoperative aberrations are more severe. Thus, in the present study, we demonstrate that VC, an important sight‐limiting HOA in KC (Erdinest et al., 2024), is more pronounced if the KC cone is decentred, and that customized CXL with delivery of high fluence over the decentred cone reduces VC to a higher extent, with indications of accompanying larger improvements in low‐contrast visual acuity. This is evidenced by the associations between LCVA 2.5% and Distance to Max and *Y*_Max_.

The reported effects of CXL on HOAs vary across studies, but the overall findings in the present study are in concordance with the existing literature. Several reports indicate a significant reduction in total HOAs following CXL treatment (Greenstein et al., 2012; Naderan & Jahanrad, 2017). Similarly, improvements in VC have been reported, at times independent from HC (Vinciguerra et al., 2013). Improvements in SA have also been documented in previous studies, albeit with varied temporal profiles (Uysal et al., 2018; Wisse et al., 2016). The present study contrasts with other previous studies, which have generally employed a uniform CXL protocol and have been unable to demonstrate larger treatment effects with larger initial HOA severity. In our material, it should be noted that a few cases had larger VC than others. However, the relationship pattern between ∆ VC, baseline VC, and *Y*_Max_ remained similar when excluding the four cases with the largest VC (dashed line in Figure 1). The correlations were significant without the four highest baseline VC at 12 months using both Pearson and Spearman correlation tests, but not at 24 months, possible due to the dropout of study participants at 24 months. Consequently, it is plausible that the substantial improvements in VC observed in these patients are attributable to their initially high baseline VC, and that the customization of the treatment patterns indeed renders different effects depending on the baseline corneal HOA status. The effect of CXL on the normal corneas in our control group also amplifies this impression. While the negative SA improved and assumed positive values from the customized treatments in KC eyes, the positive SA increased further in healthy corneas. We have shown in a previous study (Naslund et al., 2021) that a smaller treatment area can increase the rather limited effect on myopia from CXL treatment, but at the price of an increased spherical aberration. This factor limits the usefulness of CXL as a treatment for low‐grade myopia. The findings of the present study underscores the differential impact of CXL on aberrations depending on the treatment pattern and the initial corneal condition. Conversely, however, some of the highest baseline VC values in the KC group did not give rise to nearly as great improvements in VC (Figure 1). This finding itself, irrespective of its bearing on correlation results in this case, warrants further investigation into what may underlie the observed differential impact seen in cases with high pre‐treatment VC.

The effect of HOAs on visual acuity has been well‐documented although improvements in visual performance are not easily attributable to reductions in certain HOA subtypes. Seemingly, a multitude of HOAs have an impact on the overall visual function in KC. For instance, Wisse et al. identified reductions in HC as most influential on changes in uncorrected distance visual acuity (UDVA). In contrast, Greenstein et al. found no significant correlation between improvements in HOAs and UDVA or corrected distance visual acuity (CDVA) after CXL. Our present study aligns with previous findings in that a reduction in certain HOA sub‐types alone does not fully account for improvements in visual performance. Rather, it appears that a combination of aberrations interacts in determining visual quality. These results further illustrate that a nuanced understanding of how specific aberrations contribute to overall visual improvements is necessary for optimizing treatment strategies.

Although our findings need to be validated by further studies with larger sample sizes to detect smaller effects or differences with greater statistical power, the study still offers a comprehensive analysis of VC, HC and SA following customized CXL over an extended follow‐up period of 24 months. This approach provides a detailed understanding of the temporal changes in different HOAs following customized CXL treatment. This customized CXL approach seems to potentially offer better individualized results, here shown as differentiated improvements in specific HOAs. Another valuable insight is the differential effects of CXL on different corneal conditions achieved by including a control group of healthy eyes treated for low‐grade myopia.

In summary, this study corroborates previous findings that CXL can not only reduce HOAs and improve visual outcomes but also shows that the corneal re‐shaping from CXL can induce unwanted HOAs. From our present findings based on a customized CXL protocol in KC, it appears the improvements are larger in cases with higher preoperative aberrations, indicating that the concept of customization has its intended effect. The differential impact on HOA subtypes in KC and healthy corneas highlights the need for tailored treatment approaches and further investigations into the mechanisms underlying these effects.
